# NGLY1 deficiency—A rare congenital disorder of deglycosylation

**DOI:** 10.1002/jmd2.12108

**Published:** 2020-04-10

**Authors:** Patrícia Lipari Pinto, Catarina Machado, Patrícia Janeiro, Juliette Dupont, Sofia Quintas, Ana Berta Sousa, Ana Gaspar

**Affiliations:** ^1^ Pediatric Department, Santa Maria's Hospital ‐ Lisbon North University Hospital Center EPE, Pediatric University Clinic, Faculty of Medicine, University of Lisbon Lisbon Portugal; ^2^ Medical Genetics Department, Santa Maria's Hospital ‐ Lisbon North University Hospital Center EPE, Pediatric University Clinic, Faculty of Medicine, University of Lisbon Lisbon Portugal; ^3^ Metabolic Diseases Unit, Pediatric Department, Santa Maria's Hospital ‐ Lisbon North University Hospital Center EPE, Pediatric University Clinic, Faculty of Medicine, University of Lisbon Lisbon Portugal; ^4^ Neuropediatric Unit, Pediatric Department, Santa Maria's Hospital ‐ Lisbon North University Hospital Center EPE, Pediatric University Clinic, Faculty of Medicine, University of Lisbon Lisbon Portugal

**Keywords:** congenital disorders of deglycosylation, dyskinesia, global developmental delay, *NGLY1*, whole exome sequencing

## Abstract

Pathogenic variants in the NGLY1 gene are associated with a Congenital Disorder of Deglycosylation (CDDG) characterized by delays in reaching developmental milestones, complex hyperkinetic movement disorder, transient elevation of transaminases, and alacrima or hypolacrima. To date, only few cases of NGLY1 deficiency have been identified and reported in the literature. This report highlights a first child of non‐consanguineous parents with no relevant family history who presented with hypotonia and poor weight gain since birth. At 2 months, the child developed paroxysmal cervical dystonia, posteriorly resolving spontaneously by age of 3. Subsequently, delays in reaching developmental milestones, ataxia, dyskinesia, visual impairment due to cone rod retinal dystrophy, low triglycerides, and persistently elevated liver transaminases were observed. Extensive etiological investigation was performed, including array‐CGH and metabolic evaluation with no abnormalities to note. Trio whole exome analysis identified a homozygous pathogenic variant of the NGLY*1* gene, c.1891del (p.Gln631Serfs*7), consistent with CDDG. Both parents were confirmed to be heterozygous carriers. The authors discuss in this case, the clinical presentation, the diagnostic challenges, and review other relevant NGLY1 deficiency cases previously reported in the literature. This case, along with the previous reported in the literature, indicates that pathogenic variants in NGLY1 cause a recognizable phenotype and should be considered in patients with a typical presentation. It also suggests that decreased sweating is not present universally in these patients.

## 1 INTRODUCTION

Congenital Disorders of Glycosylation (CDG) are a group of inborn errors of metabolism characterized by abnormalities in biomolecules of glycosylation. Since the first description by Jaeken et al. in 1980, more than 100 different CDGs have been reported.^1^ However, to date, only one disorder of N‐linked deglycosylation has been described.^2^ NGLY1‐ Congenital Disorder of Deglycosylation (CDDG) (OMIM 610661 and 615273), is the first primary defect of N‐linked deglycosylation shown to cause human disease. Pathogenic variants in NGLY1, encoding the enzyme N‐glycanase 1, were recently found to cause a recognizable multisystemic phenotype.^3^ The first pathogenic variant was identified in 2012 by whole exome sequencing (WES) in a patient who was thought to have a Congenital Disorder of Glycosylation, but had normal serum transferrin isoelectric focusing and N‐glycan analyses on repeated tests.^3^ Since then, it has been hypothesized the cause of a multisystemic neurodevelopmental disorder in which individuals most commonly exhibit a tetrad of delays in reaching developmental milestones, hyperkinetic movement disorder, hypo/alacrima, and elevated transaminases in early childhood.^3^, ^4^, ^5^, ^6^ In recent cases described in the literature, varying lipid abnormalities such as low cholesterol,^1^, ^5^ hypotriglyceridemia, and low‐density lipoprotein were also identified.^1^

The enzyme N‐glycanase 1 removes N‐glycan species from N‐linked glycoproteins and permits degradation of misfolded proteins after translocation from the endoplasmic reticulum to the cytosol.^7^ Undegraded proteins form aggregates that may damage cells in the brain, liver and eyes, leading to the signs and symptoms of NGLY1‐CDDG.^2^

To date, according to the NGLY Foundation, approximately 63 individuals with NGLY1 deficiency have been identified.^8^ NGLY1 deficiency is caused by biallelic pathogenic variants in the NGLY1 gene, following an autosomal recessive pattern of inheritance. This article highlights the clinical and molecular findings of another patient with NGLY1 deficiency and reviews the literature describing NGLY1 deficiency in children.

## 2 CASE SUMMARY

This case describes an 8‐year‐old boy, first child of non‐consanguineous parents, born at 38 weeks of gestation (by caesarian delivery) with no known complication during pregnancy. His Apgar index was 9/10, birth weight 2650 g (10^th^ percentile), length 45 cm (third percentile), and head circumference 33.5 cm (10^th^‐50^th^ percentiles) (Fenton curves). About family history, his mother has hypercholesterolemia, with no more remarkable diseases. At his third day of life, he was admitted to the Neonatology Intensive Care Unit (NICU) due to sepsis, with apparently no identifiable agent. He was very irritable and difficult to console, with very short sleep period patterns. In the second month of life, he developed paroxysmal cervical dystonia, for which he initiated physiotherapy, with no documented improvement. Of note that, the episodes were more intense after eating and remitted when he was 3 years old.

From the fourth month onward, he failed to thrive, affecting weight more than length. On that account and on the hypothesis of cursing with gastroesophageal reflux, he was referred to a Gastroenterology Unit where liver ultrasound and oesophageal pH monitoring were performed with no abnormal results being detected. However, the upper digestive endoscopy revealed focal intestinal metaplasia. He initiated esomeprazole and domperidone, later discontinued after good clinical improvement at age 2. Still while being 2 years old, laboratory evaluation revealed persistently elevated liver transaminases (AST 91 U/L and ALT 101 U/L, reference intervals AST: 0‐34 U/L, ALT: 10‐49 U/L), low levels of triglycerides (68 mg/dL, reference interval <150 mg/dL), but normal levels of cholesterol (107 mg/dL, reference interval <190 mg/dL), and LDL (63 mg/dL, reference interval <110 mg/dL), high levels of pyruvate (0.16 mM, reference interval 0.08‐0.17), lactate (2.17 mM, reference interval 0.9‐1.8) but a normal ratio L/P (13.4, reference interval 6‐18). Given the abnormal blood test results, he was referred to Metabolic Diseases Unit for further follow‐up. At age 3, lactate (1.90 mM), pyruvate (0.15 mM) and ratio L/P (13) normalized.

During this time, the child's reflux symptoms subsided. However, his weight and height were in the third percentile when he started to present with episodes of sweating and trembling when waking up coursed with fasting, which led to the investigation of an unconfirmed hypoglycemia. Although the investigation was inconclusive, supplementation with cornstarch was added until he was 5 years old. At this time, he achieved good toleration to fasting with considerable weight gain (weight in 15^th^ percentile and height in 15^th^ percentile). By age 7, he achieved a weight in concordance with the 75^th^ percentile and height in the 10^th^ percentile. The triglycerides normalized (139 mg/dL) and levels of cholesterol (139 mg/dL) and LDL (71 mg/dL) were stable. It is noteworthy that his mother has hypercholesterolemia. The trembling resolved spontaneously, but sweating from the hands and feet persisted. This was easily identified, both, when greeting the patient, as well as, when performing the physical examination on the stretcher, in which the paper on the stretcher would get soaking wet and dripping on the floor. It was not however, possible to perform a QSWEAT/QSART test.

Due to the delays in reaching developmental milestones, he was also referred to a Neurology Unit. He sat unsupported only after 8 months of life, crawled after age one, said his first single words at age two and walked unaided after age two and a half. Using the Nijmegen CDG paediatric rating scale^9^ to determine the disease progression and severity, he currently shows a moderate impairment with a total score of 27 (6 in section I—Current Function, 2 in section II—Systemic Specific Involvement and 19 in section III—Current Clinical Assessment). He is also currently on a regular program of speech therapy and physiotherapy and has individual special education at school.

Even though, he presented, until 3 years of age, hypotonia and left lower limb dystonia, ataxia, orofacial dyskinesia, and dyskinetic movements of the upper limbs and trunk, during the following years, the dystonia and dyskinesias decreased, but orofacial and hand dyskinesia, motor coordination difficulties, dysmetria, and a wide‐based ataxic gait persisted.

On physical examination at the age of eight, his weight was in concordance with the 90^th^ percentile, height in the 25^th^ percentile and head circumference in the 10^th^ percentile. Without any striking dysmorphic features apart from small hands and feet. Lower limbs muscle atrophy and coxa valga were observed. Normal production tears with excessive sweating were also noted. Neurological examination revealed wide‐based ataxic gait, slight hypertonia of the lower limbs, orofacial, and hands dyskinesias.

The patient was thoroughly investigated by performing: ophthalmologic evaluation showing visual impairment due to cone rod retinal dystrophy; cardiac evaluation including ultrasound with no structural heart anomalies; brain‐MRI showing delayed myelination, and spectroscopy without any abnormalities. EEG displayed slow wave predominance without paroxysmal activity while muscular biopsy showed prevalence of type I fibers. Mitochondrial respiratory chain enzymatic activity had no relevant abnormalities, quantification of serum carnitine, urine organic acids, serum and urine amino acids, carbohydrate deficient transferrin, transferrin isoelectric focusing, as well as serum copper, and ceruplasmin were normal. Analysis of neurotransmitters and glucose in CSF showed nonspecific low 5‐hydroxytryptophan (105 nmol/L, RV 170‐490) and GABA (36 nmol/L, RV 60‐152). He received hydroxytryptophan supplementation without any clinical improvement. Mitochondrial DNA sequencing, molecular analysis of DGUOK and POLG1 genes, and a comprehensive NGS panel for nuclear mitochondrial genes were all normal as well as the analysis of SLC2A1 gene.

WES trio analysis allowed the identification of a homozygous pathogenic variant in the NGLY1 gene, c.1891del (p.Gln631Serfs*7), consistent with a congenital disorder of deglycosylation. Segregation analysis confirmed that both parents were heterozygous carriers of the pathogenic NGLY1 variant.

## 3 DISCUSSION

According to a database maintained by http://ngly1.org, a combination of bi‐allelic pathogenic variants in NGLY*1* gene coupled with a suggestive clinical phenotype has been identified so far, 63 individuals worldwide, with a total of 21 individuals described in the literature.^2^, ^3^, ^5^, ^6^, ^10^, ^11^, ^12^ Compound heterozygous pathogenic variants in NGLY1 gene were first reported in 2012, in a 3‐year‐old boy who presented with delay in reaching developmental milestones, multifocal epilepsy, involuntary movements, absent tears, and abnormal liver function.^3^ In 2014, Enns et al^4^ reported eight patients with bi‐allelic loss of function pathogenic variants in NGLY1. One year later, *Caglayan* et al^6^ described two children with the same phenotype, also with corneal opacities and neuropathy. In the same year, another case was reported by Heeley et al,^7^ with two novel variants in NGLY1 identified by WES (Table 1). In a study by Bosch et al,^9^ WES was performed in 25 patients with cerebral visual impairment and a visual acuity of ≤0.3, and a homozygous variant in the NGLY1 gene was detected in one patient. The phenotype of this patient was similar to previously reported patients.^9^ In 2017, six additional patients with NGLY1 deficiency were described by Lam et al, all of them with low levels of total cholesterol, low‐density lipoprotein cholesterol and triglycerides (Table 1).^1^ In 2019, two more cases have been reported in the literature, both with the intention to expand the phenotype of this disease. Chang et al^10^ report a new case of NGLY1‐CDDG with transient elevations in methionine and homocysteine, as well as S‐adenosylmethionine (SAM) and S‐adenosylhomocysteine (SAH). van Keulen et al^12^ reported a patient with homozygous mutation in NGLY1 who presented with adrenal insufficiency.

**Table 1:** Clinical and molecular findings in NGLY1 deficiency patients

Age (y)/sex	Present	1 (Need et al^3^)	2 (Enns et al^4^)	3 (Enns et al^4^)	4 (Enns et al^4^)	5 (Enns et al^4^)	6 (Enns et al^4^)	7 (Enns et al^4^)	8 (Enns et al^4^)	9 (Enns et al^4^)	10 (Caglayan et al^6^)
	8/M	3/M	5/M	20/F	4/F	2/M	5/M	9 M/F	3/F	16/F	6 M/M
*NGLY1* variants	c.1891del (p.Gln631Serfs*7)/c.1891del (p.Gln631Serfs*7)	ND	c.C1891delC(p.Q631fs)/c.1201A > T (p.R401X)	c.1370dupG(p.R458fs)/c.1370dupG (p.R458fs)	c.1205_1207del(p.402_403del)/c.1570C > T (p.R524X)	c.1201A > T(p.R401X)/c.1201A > T (p.R401X)	c.1201A > T(p.R401X)/c.1201A > T (p.R401X)	c.1201A > T(p.R401X)/c.1201A > T (p.R401X)	c.1201A > Y(p.R401X)/c.1201A > T (p.R401X)	c1201A > T(p.R401X)/c.1201A > T (p.R401X)	c.1533_1536delTCAA(p.Asn511LysfsX51)/c.1533_1536delTCAA, (p.Asn511LysfsX51)
Clinical finding											
Neurological											
GDD	+	+	+	+	+	+	+	+	+	+	+
Movement disorder	+	+	+	+	+	+	+	+	+	+	+
Hypotonia	+	ND	+	+	+	+	+	+	+	+	+
EEG abnormalities	−	ND	+	+	+	+	+	+	−	+	−
Brain MRI abnormalities	−	ND	+	−	+	+	+	+	−	+	−
Seizures	−	ND	+	−	−	−	+	−	−	+	−
Microcephaly	−	ND	−	+	+	−	+	+	+	+	ND
Peripheral neuropathy	ND	ND	+	+	ND	ND	ND	ND	ND	ND	+
Excessive sweating	+	ND	ND	ND	ND	ND	ND	ND	ND	ND	ND
Gastrointestinal											
Neonatal jaundice	−	ND	+	−	+	+	−	−	+	−	−
↑liver transaminases	+	+	+	+	+	+	+	ND	+	−	+
Low triglycerides	+	ND	ND	ND	ND	ND	ND	ND	ND	ND	ND
Liver fibrosis	ND	−	+	−	−	+	−	−	ND	ND	ND
Liver storage	ND	+	+	+	+	−	+	+	ND	ND	ND
Constipation	−	ND	+	+	+	+	+	−	+	+	ND
Ophthalmological											
Hypo/alacrima	−	+	+	+	+	+	+	−	+	+	+
Corneal ulceration	−	ND	+	+	−	+	−	−	−	+	+
Strabismus	−	ND	−	−	+	+	−	−	+	+	+
Retinal abnormalities	+	ND	ND	ND	ND	ND	ND	ND	ND	ND	−
Skeletal											
Small hands/ft	+	ND	+	−	+	+	−	−	−	+	−
Osteoporosis	−	ND	ND	ND	ND	ND	ND	ND	ND	ND	+
Scoliosis	−	ND	−	+	−	+	+	−	−	+	+
Dysmorphic features	−		−	−	−	−	+^1^	+^2^	+^3^	+^4^	−
Failure to thrive	+		−	+	−	+	+	+	−	+	ND

Age (y)/sex	11 (Caglayan et al^6^)	12 (Heeley et al^7^)	13 (Bosch et al^9^)	14 (Lam et al^1^)	15 (Lam et al^1^)	16 (Lam et al^1^)	17 (Lam et al^1^)	18 (Lam et al^1^)	19 (Lam et al^1^)	20 (Chang et al^10^)	21 (van Keulen et al^12^)
	9/F	<4/M	<4/M	16/M	5/F	6/M	8/F	10/M	17/F	5/F	8/F
NGLY1 variants	c.1533_1536delTCAA(p.Asn511LysfsX51)/c.1533_1536delTCAA, (p.Asn511LysfsX51)	c.347C > G (p.S116X)/ c.881þ5G (p.IVS5þ5G > T)	ND	c.347C > G (p.S116X)/ c.881þ5G (p.IVS5þ5G > T)	c.931G > A(p.E311K)/c.730 T > C(p.W244R)	c.1604G > A(p.W535)/c.1910delT(p.L637)	c.622C > T(p.Q208)/c.930C > T(p.G310G)	c.622C > T(p.Q208)/c.930C > T(p.G310G)	c.1201A > T(p.R401)/ c.1201A > T(p.R401)	c.1405C > T (p.Arg469*)/c.1405C > T (p.Arg469*)	c.1837del (p.Gln613fs)/c.1837del (p.Gln613fs)
Clinical findings											
Neurological											
GDD	+	+	ND	+	+	+	+	+	+	+	+
Movement disorder	−	+	+	+	+	+	+	+	+	+	ND
Hypotonia	+	+	+							+	ND
EEG abnormalities	+	+	ND	−	−	+	+	−	−	+	ND
Brain MRI abnormalities	+	−	ND	+	+	−	−	−	+	+	ND
Seizures	+	+	ND	‐(spasms)	−	−	+	−	−	−	+
Microcephaly	−	+	+	−	−	−	−	−	+	+	ND
P. Neuropathy	−	+	ND	(1)	(1)	(1)	(1)	(1)	(1)	suspected	ND
Excessive sweating	ND	ND	ND	ND	ND	ND	ND	ND	ND	ND	ND
Gastrointestinal											
Neonatal jaundice	−	+	ND	ND	ND	ND	ND	ND	ND	−	−
↑aminotransferases	+	+	ND	+	+	+	+	+	+	+	ND
↓triglycerides	ND	+	ND	+	+	+	+	+	+	−	ND
Liver fibrosis	ND	+	ND	(2)	(2)	(2)	(2)	(2)	(2)	+	ND
Liver storage	ND	+	ND	ND	ND	ND	ND	ND	ND	ND	ND
Constipation	ND	+	ND	ND	ND	ND	ND	ND	ND	+	ND
Ophthalmological											
Hypolacrima/alacrima	+	+	+	+	ND	+	+	+	ND	+	ND
Corneal ulcerations	+	−	ND	−	ND	−	−	−	+	−	ND
Strabismus	−	+	ND	+	ND	+	+	+	+	+	ND
Retinal abnormalities	−	−	ND	+	ND	+	+	+	−	−	ND
Skeletal											
Small hands/ft	−	−	ND	ND	ND	ND	ND	ND	ND	ND	ND
Osteoporosis	−	+	ND	ND	ND	ND	ND	ND	ND	+	ND
Scoliosis	−	+	ND	ND	(3)	(3)	(3)	(3)	(3)	−	+
Dysmorphic features	+^5^	+^6^	−	+^7^	+^7^	+^7^	+^7^	+^7^	+^7^	+^8^	ND
Failure to thrive	ND	ND	ND	Majority						+	ND

*Note*: 1. Mild ptosis, hypoplastic supraorbital ridges, epicanthic folds, long eyelashes, short nose, thick alveolar ridges, high‐arched palate, and mild micrognathia. 2. Epicanthic folds and short nose. 3. Frontal bossing, metopic ridge, epicanthic folds, tented upper lip, hypoplastic left nipple, tapered fingers, and mild fourth‐ and fifth‐toe clinodactyly. 4. Long eyelashes, arched eyebrows, decreased facial subcutaneous fat mild proptosis, broad nasal bridge, and crowded dentition. 5. Remarkable for corneal opacities, hypertelorism and a transverse palmar crease on her right hand. 6. Myopathic face, bilateral ptosis, hypertelorism and wide mouth. 7. Most affected individuals had hypotonic facies. The features of older individuals reflected their low weight, with thin facies, hollowed cheeks, and visible zygomatic arches. 8. Deep‐set eyes with long eyelashes, short palpebral fissures, synophrys with full, arched eyebrows, bilateral fifth finger clinodactyly and fetal pads on all fingers. (1)—8 in 12 individuals from 10 families with confirmed biallelic mutations in *NGLY1* have axonal sensorimotor polyneuropathy, but they do not specify which ones. (6 individuals were included in previous clinical publications) (2)—3 in 12 individuals from 10 families with confirmed biallelic mutations in *NGLY1* have low tryglicerides, but they do not specify which ones. (6 individuals were included in previous clinical publications) (3)—6 in 12 individuals from 10 families with confirmed biallelic mutations in *NGLY1* have scolioses, but they do not specify which ones. (6 individuals were included in previous clinical publications).

Abbreviation: ND, not determined.

The patient described in this case had similar symptoms to other clinical cases that were reported in the literature, reinforcing the notion that pathogenic variants in NGLY1 course indeed with a recognizable phenotype. NGLY1 deficiency should be suspected in individuals with the following clinical features and supportive laboratory findings: severe to profound delay in reaching developmental milestones/intellectual disability, hyperkinetic movement disorder, hypo‐ or alacrima, and elevated ALT and AST in early childhood that normalizes spontaneously, with normal transferrin glycoforms and N‐glycan profiling.^13^ The patient at hand, had several features previously associated with NGLY1 deficiency, namely, as already mentioned, global developmental delay, movement disorder, growth retardation with poor weight gain in early years, hypotonia, retinal disease, small hands and feet, low but transient triglyceride levels, and elevated liver enzymes. However, the authors could not observe nor document seizure disorder, microcephaly, hypolacrima or alacrima, peripheral neuropathy, abnormal brain imaging or osteopenia. The levels of plasma methionine were within the normal range (23.9 μM). Adrenal function was not performed; however, the patient did not show any signs of hyperpigmentation or bronze tint on physical examination. Additionally, this patient presented excessive sweating, which did not rule out the diagnosis of GLY1‐CDDG.

All types of loss‐of‐function pathogenic variants in NGLY1 gene have been reported, including nonsense, missense, frameshift, and splice site variants. The latter occur all through the gene, with no obvious hot spots.^1^ No genotype‐phenotype correlation has been observed. The pathogenic variant c.1891del (p.Gln631SerfsTer7), identified in our patient, has been previously reported^1^, ^3^, ^4^, ^14^ but only in the heterozygous form. Both parents are from Santiago do Cacém, a small municipality in the district of Setubal, in Portugal. Moreover, it was not possible to find out the carrier frequency for this variant in the geography described. The overall phenotype was attenuated in all domains (Table 1), phenomenon for which we have no explanation at this point. In the WES analysis of our case, there were no identified pathogenic/likely pathogenic variants or any variant of unknown significance that could explain the additional clinical manifestation of the case. The absence of variants in other genes, somehow, supports the possibility that the clinical phenotype may be explained by the absence of the protein in question. However, other explanations may be possible with the identification of more cases and a better understanding of the phenotype and pathophysiology of NGLY1 deficiency. There is yet another report of a sib pair sharing compound heterozygosity of cryptic pathogenic slice site variant (c.930C > T) and a nonsense variant (p.Gln208Ter) in the NGLY1 gene, who both exhibited relatively mild impairment in all domains.^1^ So far, there is no analysis of functional activity of the different genetic variants in the NGLY1 gene, which could be helpful to establish a correlation between genotype‐phenotype.

Currently, there are no FDA‐approved treatments for NGLY1 deficiency. Enzyme replacement therapy is currently being evaluated and, recently, a proposed molecular mechanism for NGLY1 deficiency suggested that endo‐β‐N‐acetylglucosaminidase (ENGase) inhibitors may be a promising therapy for NGLY1 patients.^15^ Regarding therapies, the authors refer only some Proton Pump Inhibitors such as Lansoprazole, Rabeprazole, Omeprazole, Dexlansoprazole, and Tenatoprazole. Our patient was previously treated with Esomeprazole, also an ENGase inhibitor, in the first years of age with improvement of his digestive symptoms. Highlighting the fact that patient had gastroesophageal reflux and the short period of therapy, it is nevertheless interesting the observed patient clinical improvement with this therapy, establishing a potential causal relationship.

In summary, we present a patient with NGLY1 deficiency with a homozygous frameshift pathogenic variant detected by WES. This case, along with previously reported cases, in the literature, indicates that pathogenic variants in NGLY1 gene cause a recognizable phenotype and should be considered in patients with a typical presentation.

This report is also intended to raise awareness about the typical phenotype, which in turn is expected to result in earlier diagnosis and a reduced number of exams and analytical investigations.

## CONFLICT OF INTEREST

The authors declare no conflicts of interest.

## AUTHOR CONTRIBUTIONS

P.L.P.: substantial contributions to the drafting, conception and design of the work, the acquisition, analysis, and interpretation of data for the work. C.M.: substantial contribution to the conception, design, and revise of the work critically for important intellectual content. J.D.: substantial contribution to the conception, design, and revise of the work critically for important intellectual content. P.J.: substantial contribution to the planning, conception, design, acquisition, analysis, and interpretation of data for the work and also revise of the work critically for important intellectual content. S.Q.: substantial contribution to the analysis, and interpretation of data for the work and also revise of the work critically for important intellectual content. A.B.S.: substantial contribution to the design and revise of the work critically for important intellectual content. A.G.: substantial contribution to the conception, design, and revise of the work critically for important intellectual content. All the authors also: approve the version to be published and agree to be accountable for all aspects of the work in ensuring that questions related to the accuracy or integrity of any part of the work are appropriately investigated and resolved.

## INFORMED CONSENT

All procedures followed were in accordance with the ethical standards of the responsible committee on human experimentation (institutional and national) and with the Helsinki Declaration of 1975, as revised in 2000. Informed consent was obtained from the patient for being included in the article.

## ANIMAL RIGHTS

This article does not contain any studies with human or animal subjects performed by any of the authors.

## DETAILS OF ETHICS APPROVAL

Ethics committee approval not applicable.
